# Vaccination hesitancy among women of reproductive age in resource-challenged settings: a cause for public health concern

**DOI:** 10.11604/pamj.2021.38.336.28953

**Published:** 2021-04-07

**Authors:** Grant Murewanhema

**Affiliations:** 1 Unit of Obstetrics and Gynaecology, Department of Primary Health Sciences, Faculty of Medicine and Health Sciences, University of Zimbabwe, Harare, Zimbabwe

**Keywords:** COVID-19, SARS-CoV-2, vaccines, hesitancy, women of reproductive age

## Abstract

Women of reproductive age are a key population for the control of COVID-19 owing to their levels of socio-economic activities and central role in the upkeep of stable families. Therefore, adequate vaccination uptake in this population is critical. However, this may be negatively affected by circulating rumours regarding SARS-CoV-2 vaccines and subfertility and pregnancy and breastfeeding concerns that have circulated widely on diverse social media. Urgent public health interventions are required to deal with vaccination hesitancy and promote uptake in this key population. This calls for concerted, multidisciplinary and multistakeholder consultative public health forums, public health research and action in the shortest possible period.

## Perspective

From March 2020 when the COVID-19 global outbreak was declared a pandemic [1], the crux of containing the spread revolved around infection prevention and control protocols dependent on human willingness and compliance. In December 2020, the World Health Organisation (WHO) began emergency approvals for SARS-CoV-2 vaccines [2]. This brought hope of another layer of protection, which is not so much dependent on human behaviour but uptake. Vaccination has been one of public health´s greatest success stories for reducing the burden, morbidity and mortality associated with infectious diseases [3]. An effective primary prevention strategy can substantially reduce the strain on healthcare resources imposed by fast-spreading epidemics and pandemics [3].

Uptake is the key component of success for any vaccination programme. Achieving herd immunity requires a threshold proportion of the population to have been vaccinated [4,5], to realise benefits at population level. This is estimated to be around 67% for SARS-CoV-2 vaccines [5,6]. Unfortunately, COVID-19 vaccination programmes globally face a high level of vaccination hesitancy [6,7]. This is defined by the WHO as a delay in acceptance or total refusal despite availability of vaccination services [8]. It is a complex process where several factors interplay, dependent on cultural and socioeconomic contexts. The WHO vaccine hesitancy model aggregates drivers of vaccine hesitancy into complacency, confidence and convenience factors [8]. In low-resource contexts such as those in sub-Saharan Africa, several factors have arisen driving the hesitancy and propagated by widespread dissemination across different social media [4]. Themes centred on vaccine fear, mistrust, religion, myths and misconceptions and conspiracy theories have emerged and are promoted by a huge gap in communication by responsible governments and public health authorities [5]. Different populations across the divide may have different perspectives and attitudes towards SARS-CoV-2 vaccines and may have different determinants of uptake. Public health stakeholders must take into account the different population needs and dynamics when designing effective strategies to improve the uptake of the vaccines, informed by contextualised research.

Women of reproductive age (WRA) are a unique population who consider not just themselves but also their current and future fertility when making decisions regarding vaccine uptake. By definition, WRA refers to all women between 15 and 49 years of age. They are a key population for pandemic control because they are socioeconomically very active, mobile and look after their families. They may therefore contribute immensely to the spread of SARS-CoV-2, but their being affected by the pandemic can also result in instability and social challenges for their families. What they read and follow on social media and learn from their peers may also have a huge influence on these decisions. We discuss factors unique to WRA that may have a negative influence on vaccine uptake and give recommendations to mitigate against these.

**Vaccination hesitancy factors unique to women of reproductive age:** factors unique to WRA centre on future fertility, current pregnancy and breastfeeding. Widespread rumours, myths and misconceptions have been circulated widely on WhatsApp, Twitter and Facebook that SARS-CoV-2 vaccines will result in subfertility [9]. The origin of this is unclear, but this may a have a remarkable negative impact of vaccination uptake in the African context. Childbearing cements many African families [10], where children have traditionally been viewed as a symbol of wealth and success and have been used to provide labour for various socioeconomic activities. Children secure conjugal ties, offer social security and maintain the lineage name. Some religious entities, especially of the apostolic sects, widespread in Africa, place a central role on childbearing and dissuade their congregants from taking contraceptives, in order to be ‘fruitful and multiply´ [11]. Sadly, in this context, conception failure is traditionally blamed on the woman and is a potential source of conflict, separation or polygamy, all disadvantaging the WRA. Therefore, anything perceived to have a negative impact on childbearing may not be well accepted and the circulating myth associating vaccines and subfertility may prevent their successful uptake.

Current vaccines are not yet been adequately tested in pregnancy and their effects on the developing foetus are unknown. There is currently no evidence that the current SARS-CoV-2 vaccines are harmful in pregnancy. However, clinical trials are in the pipeline that will determine their safety profiles in pregnancy. Meanwhile, several messages have been distributed across different social media linking vaccines to miscarriages, giving birth to children with abnormalities and other adverse outcomes. No mother would want to lose their unborn child and some dread the trauma of having to look after a child with disability. Many mothers in resource-limited settings are aware of a family that looks after a child with a disability, such as cerebral palsy, or Down´s syndrome and would never want a similar experience. Some developed countries that commenced vaccination earlier discouraged or advised breastfeeding women against being vaccinated, without any concretely supporting evidence [12]. Theoretically, COVID-19 vaccines could be beneficial to a breastfeeding child due to transfer of antibodies, providing passive immunity. However, this is not yet adequately explored. Mothers are heavily protective of their babies and the circulating misconception that vaccinating breastfeeding mothers can harm their babies may negatively influence the uptake of vaccines by WRA. Breastfeeding mothers willing to be vaccinated must be encouraged and supported as they may have considered their risk versus the benefit of vaccination. Concerns regarding the safety of SARS-CoV-2 vaccines during pregnancy and breastfeeding must be urgently addressed, given that some WRA work in high-risk frontline jobs [12]. A substantial proportion of nurses and other healthcare workers are females who are in the WRA category. Vaccinating these frontline workers not only protects them, but also their clients. Others may also suffer from chronic diseases, making them vulnerable and would benefit immensely from the protection conferred by safe and effective vaccines. Apart from healthcare workers, WRA also constitute significant proportions in other sectors such as education and security and may potentially be at high risk of contracting SARS-CoV-2 from their students and clients.

**Understanding the drivers of vaccination hesitancy among women of reproductive age:** effectively dealing with vaccination hesitancy premises upon good understanding of the barriers. There is need for more directed research aimed at exploring these barriers and seeking insights into the possible promoters. Reinforcing mechanisms are critical for those who are vaccine accepters and mitigating solutions aimed at those against vaccine uptake are needed. Thus, critical thinking to design innovative solutions is desirable. Socioeconomic and cultural contexts differ; therefore, there is no single solution for all. Whilst the social media has been largely viewed as a source of vaccination hesitancy in many instances, situation-specific contexts must be explored adequately. In the HIV era, ethnographic studies were an important source for understanding the drivers of transmission among young females [13]. Similarly, qualitative studies to explore the different contexts through in-depth interviews and focus group discussions potentially can provide important insights into vaccination hesitancy. Behavioural scientists are an integral part of public health and must take the lead in this drive.

Understanding and following trends on social media helps to identify common emerging themes [14,15]. Messages by influential religious leaders, antivaxxers and conspiracy theorists tend to be widely circulated on social media and because of the explosion of WhatsApp and other social media usage, they tend to go viral within a very short space of time [15]. A pro-active approach to dealing with these messages is required. Thus, stakeholders in public health must continue designing ways of timeously identifying circulating falsehoods and responding appropriately in time. Conspiracy theories also rapidly evolve and public health is often found lagging behind.

**The central role of public health in addressing vaccination hesitancy among women of reproductive age:** public health entities must work closely with relevant authorities to promote vaccination uptake in their spheres of influence. There is need for an integrated, interdisciplinary approach that takes into account the diverse contexts of WRA, to design and implement effective uptake strategies. Vaccination hesitancy is a big global health threat and requires urgent concerted redress from all relevant stakeholders [7]. Public health practitioners have a central role in carrying out relevant research to answer questions of vaccine safety and effectiveness in WRA and address the concerns regarding subfertility, pregnancy and breastfeeding. The other important role is to generate evidence-based information, education and communication material suitable for different contexts and in different languages and communicate these effectively and timeously. The heavy presence of WRA on social media brings a tremendous opportunity to widely disseminate correct messages and counter the various circulating falsehoods, myths and misconception [15]. The communication vacuum left by governments and partners as they concentrate on COVID-19 response and containment activities has provided a gap for conspiracy theorists to disseminate potentially detrimental information. Therefore, players in public health must urgently bridge this gap.

## Conclusion

Addressing vaccination hesitancy among WRA is an urgent public health priority. Relevant stakeholders in public health must urgently find ways of addressing concerns regarding the safety of vaccination in this population concerning fertility, pregnancy and breastfeeding. In this regard, a multidisciplinary, multistakeholder approach is critical to achieve success, as we work towards avoiding further waves and devastating consequences of COVID-19 in resource-challenged settings.
